# Cardiovascular and Metabolic Crosstalk in the Brain: Leptin and Resistin

**DOI:** 10.3389/fphys.2021.639417

**Published:** 2021-02-18

**Authors:** Emilio Badoer

**Affiliations:** School of Health and Biomedical Sciences, RMIT University, Melbourne, VIC, Australia

**Keywords:** leptin, resistin, central nervous system, sympathetic nerve activity, metabolic syndrome, interactions

## Abstract

Leptin and resistin are cytokines whose plasma levels correlate with adiposity. Leptin is a hormone synthesised and released from adipocytes and can be transported into the brain. Resistin is produced in adipocytes in rodents and in macrophages in humans, particularly macrophages that have infiltrated adipose tissue. Both hormones can act within the brain to influence sympathetic nerve activity. Leptin appears to have a generalised sympatho-excitatory actions whilst resistin appears to increase sympathetic nerve activity affecting the cardiovascular system but inhibits sympathetic nerve activity to brown adipose tissue, which contrasts with leptin. Since both hormones can be elevated in conditions of metabolic dysfunction, interactions/crosstalk between these two hormones in the brain is a real possibility. This review describes the current knowledge regarding such crosstalk within the central nervous system. The evidence suggests that with respect to sympathetic nerve activity, crosstalk between leptin and resistin can elicit enhanced sympatho-excitatory responses to the kidneys. In contrast, with respect to food intake, resistin has weaker effects, but in regard to insulin secretion and thermogenesis, leptin and resistin have opposing actions. Thus, in conditions in which there is increased resistin and leptin levels, the result of crosstalk in the central nervous system could contribute to worse cardiovascular and metabolic complications.

## Introduction

In conditions like metabolic syndrome, overweight/obesity and Type 2 diabetes, there is increased body weight, insulin resistance, hyperglycemia, hypertension, and metabolic dysfunction including elevated levels of leptin and resistin. Both these hormones have central and peripheral actions. This review will focus on the central actions of these hormones on metabolic function and cardiovascular regulation and discusses what is known of interactions/crosstalk between leptin and resistin, particularly in relation to cardiovascular and metabolic regulation.

### Leptin

It was well known that lesions of the hypothalamus can result in obesity and the critical parabiotic studies using the *ob/ob*, *db.db* and lean control mice in the mid-60s led investigators to hypothesise a circulating factor that was a key regulator of metabolism. In 1994, leptin was first identified by Friedman as a circulating factor originating from fat tissue that was able to regulate energy balance (Zhang et al., 1994). A short but fascinating insight into the discovery of leptin is provided by Friedman in his Harrington Prize essay (Friedman, 2016). Although the amount of fat tissue in the body correlates with the level of circulating leptin, suggesting that white adipose tissue is the main producer of leptin, other tissues have also been found to produce the hormone, such as liver, stomach and even brain (Wang et al., 1998).

Leptin is a 16 kDa polypeptide which is able to cross the blood brain barrier courtesy of a carrier transport system. The leptin-receptor mediated carrier transport occurs mainly in the choroid plexus that lines the walls of the cerebral ventricles and in circumventricular organs like the median eminence and lamina terminalis. Some studies, predominantly using radiolabelled leptin, have suggested that altered transport of leptin across the blood brain barrier accounts for the resistance to the metabolic actions of leptin that is characteristic of diet-induced obesity (Banks et al., 1999). However, recent studies using fluorescently labelled leptin, suggests that the transport of leptin into the brain is not affected by dietary-induced obesity (Harrison et al., 2019). Note that neurons positive for agouti-related peptide and pro-opiomelanocortin in the arcuate nucleus (ARC) also send their dendritic projections into the median eminence and contain leptin receptors allowing direct access of circulating leptin to those neurons (Ha et al., 2013).

The actions of leptin are mediated by specific receptors. There are several isoforms of the receptor due to alternate splicing of the mRNA and the products have been grouped into long, short and secretory forms of the receptor. It is the long form that has the capability of inducing all the known intracellular transductions pathways associated with leptin receptor activation and is primarily found in the hypothalamus of the brain. The short form is believed to be associated with transmembrane transport of leptin into cells and the secretory form is believed to act as a circulating carrier for leptin (Tartaglia, 1997).

### Metabolic Actions of Leptin

Metabolic homeostasis is determined by the simple equation; intake vs expenditure. The amount of food we ingest and the amount of energy we expend determines our metabolic balance. Ingesting more food than the energy we expend will see the balance tip towards weight gain and vice versa. Leptin is a key regulator of both sides of the equation. The amount of leptin circulating correlates with the amount of fat, so as we ingest more food and build up our fat stores leptin levels will increase. Leptin acts within the brain as an anorectic agent to reduce food intake; a classic negative feedback.

The actions of leptin may be affected by ambient temperature. This is most evident in animals like tree shrews (*Tupaia belangeri*) and Brandt’s vole (*Lasiopodomys brandtii*) that acclimate to a cold environment by increasing body mass and energy expenditure. In such cases plasma leptin levels are low in contrast to conditions in which rewarming takes place where body mass and energy expenditure drop and plasma leptin levels increase (Zhang and Wang, 2008; Zhang et al., 2012).

The key nuclei in the brain in which leptin influences dietary intake are located primarily within the hypothalamus. Leptin activates neurons located in the lateral ARC that are positive for proopiomelanocortin (POMC)/cocaine- and amphetamine-regulated transcript (CART) and reduces the activity of agouti-related peptide (AgRP)/neuropeptide Y (NPY)/GABA-containing neurons located more medially in the ARC (Cowley et al., 2001; Abizaid et al., 2006). Note that leptin receptors are predominantly, but not exclusively present, on these neurons (Cowley et al., 2001).

Direct projections from the ARC to other hypothalamic nuclei play key roles in mediating the metabolic effects of Leptin. The ARC projects directly to the paraventricular nucleus, a key neuroendocrine and integrative nucleus, and ARC neurons also project directly to the dorsomedial hypothalamus (DMH). As indicated earlier, leptin receptors are present on POMC/CART and on NPY/AgRP/GABA-containing neurons within the ARC which would suggest that the pathways involved in mediating leptin’s anorectic effects involve activation of the POMC/CART neuronal population that projects to the hypothalamic paravemtricular nucleus (PVN) and the DMH which results in activation of melanocortin receptors by the neurotransmitter, alpha-melanocyte stimulating hormone (MSH) released from the POMC/CART neurons, and inhibition of the NPY/AgRP/GABA neurons. However, this is clearly not the full story since knockdown of leptin receptor-positive POMC neurons only leads to mild obesity (Balthasar et al., 2005). Similarly, knockdown of Leptin receptor positive NPY/AgRP/GABA neurons in the ARC cannot fully explain the effects of leptin on dietary intake since only mild obesity also results in such mice (Tong et al., 2008). These findings suggest that leptin may influence ARC neurons containing POMC or those containing AgRP indirectly as well as indirectly to inhibit dietary intake.

Eating a meal can provide gustatory pleasure, thus reward and desire can play major roles in dietary intake. Leptin may influence these pathways to modify dietary intake. The reward pathways in the brain involve the mesolimbic dopaminergic pathways from the ventral tegmental area (VTA). Leptin receptors are present in the VTA but knockdown of the leptin receptor-positive dopaminergic neurons in the VTA did not appear to influence motivational food-seeking behaviour but did increase anxiety (Liu et al., 2011). However, the deletion of VTA neurons containing the leptin receptor does markedly influence dietary intake and body weight (Hommel et al., 2006). These findings suggest that in relation to food seeking behaviour, the VTA dopaminergic system is not directly influenced by leptin. However, indirect effects of leptin via neurons that contain leptin receptors and project from the lateral hypothalamic area to the VTA may have a key role, since deletion of such lateral hypothalamic neurons can increase food intake and weight gain (Leinninger et al., 2011).

Leptin actions in the brain also induce increased energy expenditure. These effects involve the neuroendocrine and autonomic nervous systems, and the hypothalamic PVN plays a critical role. The PVN contains neurons that synthesise and release thyroid stimulating hormone (TSH) and corticotrophin (CRH). TSH acts upon the thyroid gland to enhance thyroid hormone production which ultimately can lead to increased basal metabolic rate and increased energy expenditure (Flier et al., 2000). The PVN also has the anatomical connections to directly and indirectly influence the sympathetic preganglionic motoneurons and thus sympathetic nerve activity (Shafton et al., 1998). Centrally administered leptin increases sympathetic nerve activity to brown adipose tissue resulting in increased non-shivering thermogenesis (Rahmouni et al., 2009). Induction of thermogenesis in brown adipose tissue has the potential to expend incredible amounts of energy since reports show that activation of 40–50 g of brown adipose tissue in humans could result in a 20% increase in energy expenditure (Rothwell and Stock, 1983). The central mechanisms involved have recently been shown to involve brain-derived neurotrophic factor (BDNF)-containing neurons within the PVN. This beautiful work has shown a critical role of BDNF in manipulating synaptic development and connectivity and in mediating the effects of leptin. In essence leptin by influencing the activity of the POMC and AgRP neurons within the ARC that project to the PVN drive increased thermogenesis by increased sympathetic nerve activity to brown adipose tissue (Wang et al., 2020). Incredibly, Not only is sympathetic nerve activity influenced, but the degree of sympathetic innervation of brown adipose tissue is affected such that chronically administered leptin into the cerebral ventricles of the brain increased sympathetic nerve terminals in brown adipose tissue, whilst *ob/ob* mice (i.e., lack functional leptin) has significantly reduced innervation of brown adipose tissue and correlates with their reduced thermogenic responsiveness to cold (Wang et al., 2020).

### Cardiovascular Actions of Leptin

Leptin, acting within the brain, can increase blood pressure in pre-clinical studies and it can contribute to the hypertension associated with obesity (Lim et al., 2013; Simonds et al., 2014, 2017). In clinical studies approximately 50% of the link in blood pressure and body weight has been estimated to be due to the changes in the levels of leptin (Abramson et al., 2011). The mechanisms involved are not fully understood, but an increase in sympathetic nerve activity undoubtedly contributes (Lim et al., 2013; Simonds et al., 2014, 2017). Leptin induces a generalised increase in sympathetic nerve activity following acute intracerebroventricular administration. The increases in sympathetic nerve activity include the efferent outflow to the kidney, skeletal muscle vasculature, splanchnic vasculature, adrenal gland, and also brown adipose tissue (Dunbar et al., 1997; Haynes et al., 1997). The increase in renal sympathetic nerve activity may play an important role in the long-term action of leptin on blood pressure (Lim et al., 2013). The central pathways utilised by leptin to elicit the sympatho-excitatory responses are likely to be include the same nuclei to those used for leptin’s metabolic actions.

It is also noteworthy that peripheral sites of action can also contribute to leptin’s cardiovascular actions, particularly recent work which describes a key role for circulating leptin acting within the carotid body (Shin et al., 2019). In that study, intravenously administered leptin increased blood pressure in lean mice and was prevented by carotid body denervation. The increase in blood pressure that was elicited by the activation of leptin receptors was mediated by TRPM7 calcium channels. It was also found that the selective overexpression of the leptin receptor in the carotid body in mice deficient of the leptin receptor enhanced TRPM7 gene expression and induced hypertension (Shin et al., 2019). This suggests that there may be some complex interplay in cardiovascular effects between peripherally- and centrally acting leptin and perhaps environmental stimuli. Future work should address this.

### Transduction Pathways Utilised by Leptin

The intracellular transduction pathways involved in the metabolic and sympatho-excitatory responses induced by leptin involve PI 3-Kinase, ERK1/2, and MTORC1 (Rahmouni et al., 2003, 2009; Kim et al., 2006; Plum et al., 2006). However, the contributions of each of these may differ between end organs. For example, the increase in sympathetic nerve activity to the kidneys involves activation of PI 3-kinase (Rahmouni et al., 2003, 2009), but this is not the case for lumbar and adrenal sympathetic nerve activities. Furthermore, sympathetic nerve activity subserving brown adipose tissue involves ERK 1/2 (Rahmouni et al., 2009).

## Resistin

Resistin is a member of the resistin-like molecule hormone family which is characterised by 10 conserved cysteine residues and a conserved cysteine residue located near the amino terminal end (Steppan et al., 2001; Steppan and Lazar, 2002). Resistin is secreted as a disulphide-linked homotrimer and circulates in plasma as such or a hexamer (Ghosh et al., 2003). In rodents, white adipose tissue is the main source of synthesis and secretion of resistin and its expression differs depending on the location of the adipose tissue and also with gender (Steppan et al., 2001). In humans, the main source of resistin is macrophages and in obesity it is the macrophages that have infiltrated into visceral white adipose tissue (Yang et al., 2003; Curat et al., 2006). This is likely to be the reason that resistin levels in plasma are reported to be elevated in obesity and diabetes (Steppan et al., 2001; Azuma et al., 2003; Rajala et al., 2004). Resistin may also be synthesised in other tissues and organs including the brain including the ARC, ventromedial nucleus and hippocampus (Morash et al., 2002; Wilkinson et al., 2005). Resistin has been detected in cerebrospinal fluid of humans.

It has been almost 20 years since resistin was first discovered, yet its specific receptors have not been identified. Several candidates have been suggested and these include a metabolite of of a proteoglycan known as decorin. This may be involved in growth of white adipose tissue (Daquinag et al., 2011). Adenyl cyclase associated protein-1 which may contribute to inflammatory processes in monocytes (Lee et al., 2014), and toll like receptor 4 (Tarkowski et al., 2010). The latter two would appear candidates for mediating the pro-inflammatory actions that have been ascribed to resistin. A fourth candidate that has been suggested in the mouse is receptor tyrosine kinase-like orphan receptor 1 (ROR1) (Sanchez-Solana et al., 2012).

### Metabolic Actions of Resistin

Resistin was originally identified due to its association with insulin resistance (hence its name) (Steppan et al., 2001; Steppan and Lazar, 2002). Of particular interest is that in rodents, resistin induces insulin resistance in the metabolically important organ, the liver (Sheng et al., 2008). The plasma levels of resistin levels are increased in obesity and diabetes and several human studies indicate a relationship between plasma resistin levels and increased obesity (Steppan et al., 2001; Azuma et al., 2003; Rajala et al., 2004). Although the majority of studies confirm the correlation between resistin and obesity and Type 2 diabetes, not all studies show increases in resistin levels (Iqbal et al., 2005; Lee et al., 2005). Thus, the role of resistin in the pathology of obesity and the development of Type 2 diabetes still requires clarification. Nonetheless, clinical studies support the use of plasma levels of resistin as a biomarker for conditions such as inflammation, cancer, atherosclerosis and cardiovascular disease (Filkova et al., 2009).

Resistin has been reported to elicit acute reductions in dietary food intake (Tovar et al., 2005) but this observed with chronic administration (Park et al., 2008). Resistin acts centrally to decrease energy expenditure. Following central administration of resistin there is a reduction in sympathetic nerve activity to brown adipose tissue (Kosari et al., 2011) which contrasts directly with the actions of leptin. Brown adipose tissue is a key organ responsible for non-shivering thermogenesis (Cannon and Nedergaard, 2004). Uncoupling protein-1 is located on the inner mitochondrial membrane of brown adipose cells and its role is to uncouple oxidation from ATP synthesis resulting in a marked increase in heat production (Cannon and Nedergaard, 2004). Brown adipose tissue was thought to reduce with age in humans but it is now recognised that depots of functionally active brown adipose tissue exist in adult humans (Nedergaard et al., 2007). The thermogenic function of brown adipose tissue is tightly regulated by the sympathetic nervous system. Thus, resistin acting within the brain has been shown to decrease body core temperature and this correlates with a reduction in the temperature of brown adipose tissue and is mediated by the reduction in sympathetic nerve activity to brown adipose tissue (Kosari et al., 2013). The data suggests resistin has inhibitory actions on thermogenesis and could exacerbate metabolic complications in conditions like metabolic syndrome and Type 2 diabetes.

### Cardiovascular Actions of Resistin

Several clinical studies have reported correlations between plasma resistin levels and high blood pressure (Papadopoulos et al., 2008, 2009; Thomopoulos et al., 2010; Zhang et al., 2010). Additionally, the levels of resistin in plasma may have a predictive value since it has been reported that in a young healthy population with a family history for essential hypertension, the levels of resistin in plasma were increased (Papadopoulos et al., 2008). This view is supported by observations that the risk of developing hypertension over a 14 year follow up of 872 women without previous history of hypertension or diabetes positively correlates with plasma resistin levels (Zhang et al., 2010). Nonetheless, the contribution of resistin to hypertension still remains controversial as there are few studies that have not observed a significant correlation, however, other risk factors may also contribute such as the presence of Type 2 diabetes (Asgary et al., 2014).

The mechanisms that could contribute to elevations in blood pressure are changes in sympathetic nerve efferent outflow. Acute intracerebroventricular administration of resistin elicits significant increases in sympathetic nerve activity including that to the skeletal muscle vasculature and kidneys (Kosari et al., 2011, 2012). It is well documented that sympathetic nerve activity to the muscle vasculature and to the kidneys is increased in obesity and diabetes, and this could suggest that resistin may contribute to the cardiovascular complications associated with conditions of metabolic dysfunction, as has been found for leptin. This requires further investigation.

Resistin does not induce a generalised increase in sympathetic nerve activity, rather it appears that sympathetic nerve efferent outputs to organs/tissues that are important in cardiovascular regulation are increased but sympathetic nerve activity to metabolically important tissue (e.g., brown adipose tissue) is reduced. Thus, the sympathetic efferent pathways influencing cardiovascular outputs are increased by both resistin and leptin. In contrast, the metabolic sympathetic efferent outputs elicited by resistin opposes the effects of leptin.

### Transduction Pathways Utilised for the Cardiovascular and Metabolic Actions of Resistin

The intracellular mechanisms involved in the transduction of the renal sympathetic nerve responses elicited by centrally administered resistin is mediated by PI 3-kinase (Kosari et al., 2012). The central sites directly activated by resistin are not yet clearly elucidated. However, the paraventricular nucleus in the hypothalamus is likely to be a major site (Tovar et al., 2005; Singhal et al., 2007; Badoer, 2010; Kosari et al., 2011). This nucleus plays a key role in renal sympathetic nerve responses mediating body fluid regulation (Ng et al., 2004).

The intracellular transduction mechanisms mediating the central responses to resistin on sympathetic nerve activity to brown adipose tissue involves ERK 1/2 dependent pathways. However, the inhibition of PI 3-kinase did not influence the sympatho-inhibitory action of resistin (Kosari et al., 2012). Thus, the intracellular transduction pathways utilised by resistin in the brain mediating the sympatho-excitatory effects to the kidneys and the sympatho-inhibitory responses to brown adipose tissue are different.

### Crosstalk in the Central Nervous System

As we have seen earlier, leptin and resistin act centrally to increase sympathetic nerve activity to the kidneys and skeletal muscle vasculature (Rahmouni et al., 2003; Kosari et al., 2011, 2012). In contrast, resistin acts centrally to inhibit sympathetic nerve activity to brown adipose tissue (Kosari et al., 2011), whilst leptin increases sympathetic nerve activity to this tissue (Rahmouni et al., 2009). These findings suggest that there is the potential for central interaction/crosstalk between these two hormones in cardiovascular and metabolic regulation. This could have important implications since plasma leptin and resistin levels correlate with adiposity (Shimizu et al., 1997; Steppan et al., 2001; Azuma et al., 2003; Rajala et al., 2004), and both can be elevated in obesity and overweight conditions.

### Crosstalk and Cardiovascular Regulation

**FIGURE 1:**
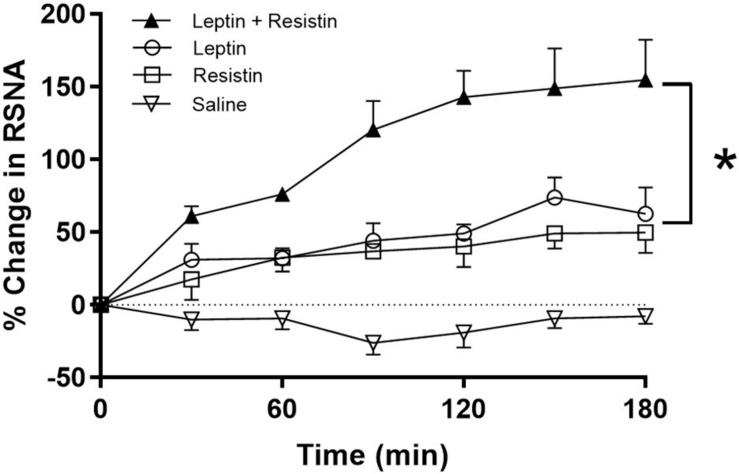
% Change in renal sympathetic nerve activity (RSNA) over time following intracerebroventricular administration of vehicle, resistin, leptin, alone and in combination. Note the enhanced increase in RSNA when leptin and resistin were administered in combination. Modified from Habeeballah et al. (2016). * represent significantly greater than leptin or resistin administered alone.

**FIGURE 2:**
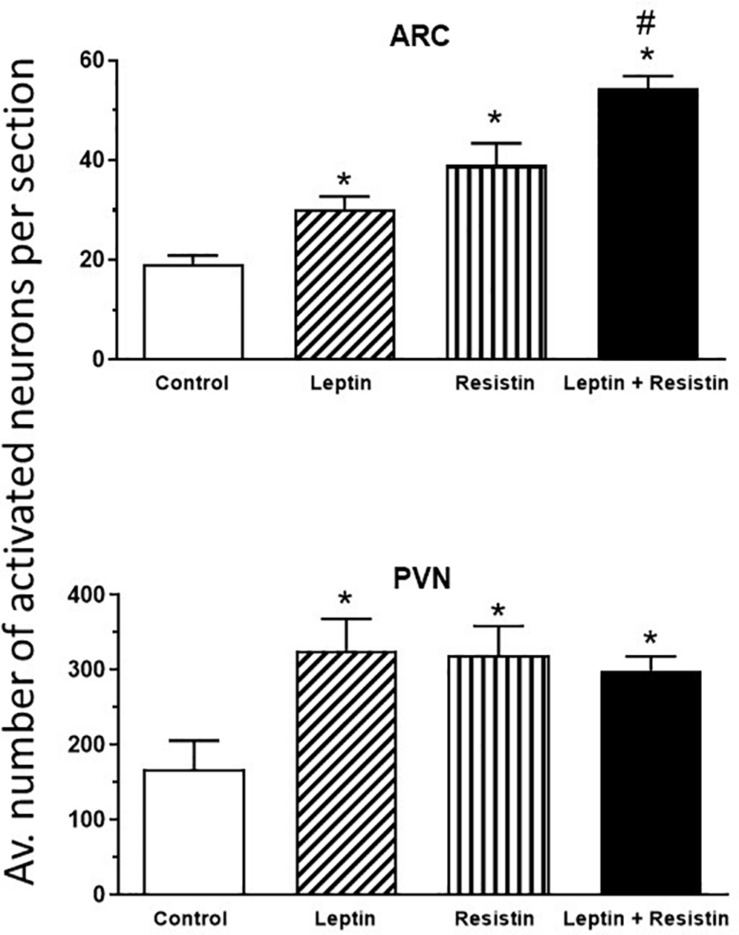
Average number of activated neurons per section in the arcuate nucleus (ARC) and paraventricular nucleus (PVN) in the hypothalamus in control or following administration of leptin or resistin alone or in combination. Modified from Kosari et al. (2012). * represent significantly greater than control. # represents significantly greater than leptin or resistin administered alone. Note that only in the ARC was there a greater number of activated neurons following leptin and resistin combined compared to when each was administered alone.

The potential central crosstalk between leptin and resistin in the regulation of blood pressure, heart rate and sympathetic nerve activity to the kidney was recently investigated. There was no evidence of crosstalk in blood pressure or heart rate effects. However, when resistin was administered intracerebroventricularly 15 min after leptin the increase in renal sympathetic nerve activity was at least double that observed with each hormone alone (Figure 1) suggesting an additive effect or even perhaps a synergistic effect (Habeeballah et al., 2016). Resistin also induced a significant increase in the number of activated neurons, identified as neurons that contain the immediate early gene protein Fos, in the arcuate, and paraventricular nuclei in the hypothalamus (Singhal et al., 2007; Kosari et al., 2011), as has been observed with leptin administration (van Dijk et al., 1996; Woods and Stock, 1996; Elias et al., 2000), When leptin and resistin were administered in combination, the numbers of Fos-positive cell nuclei (a marker of activated neurons) in the arcuate and in the paraventricular nuclei were significantly greater than in controls, but only in the ARC was the increase significantly greater than either hormone alone (Figure 2). The findings suggest that in the ARC there may be separate populations of neurons activated by leptin compared to resistin. However, in the paraventricular nucleus the results suggest that the same population of neurons was activated by leptin or resistin, because similar numbers of neurons were activated in that brain nucleus following leptin and resistin alone or in combination (Figure 2). This could be explained if the same neuronal population was activated to a much higher level by the administration of leptin and resistin combined than that observed after the administration of either hormone alone. Note, that Fos is used to identify increased activity in neurons but does not provide a measure of the degree of increased neuronal activity.

The sympatho-excitatory action of leptin on renal sympathetic nerve activity is not affected by high fat diets. However, diets high in fat lead to a reduction in the sensitivity to the anorexigenic actions of leptin (Widdowson et al., 1997; El-Haschimi et al., 2000). This selective resistance to leptin (Correia et al., 2002; Rahmouni et al., 2002, 2005; Mark, 2013) could be a key contributor to obesity-induced hypertension (Prior et al., 2010; Eppel et al., 2013).

**FIGURE 3:**
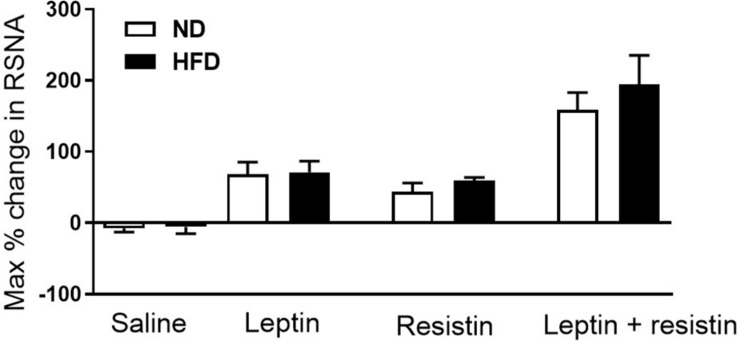
Maximum% change in renal sympathetic nerve activity in rats fed normal chow or a moderately high fat diet (22% fat) following intracerebroventricular saline, leptin or resistin alone or in combination. Note there was no influence of the diet. Modified from Habeeballah et al. (2017).

Whether a high fat diet could influence the cardiovascular effects of resistin and the crosstalk between leptin and resistin has been investigated. A moderate high fat diet (22% fat) which induced a 40% increase in whole body fat mass did not influence the blood pressure or heart rate responses to leptin and resistin combined (Habeeballah et al., 2017). The sensitivity to the sympatho-excitatory effects on renal sympathetic nerve activity produced by resistin was not affected by high fat feeding, which has also been observed with leptin (Rahmouni et al., 2005; Carroll et al., 2006; Morgan et al., 2008; Habeeballah et al., 2017). In the rats fed the high fat diet, leptin and resistin combined elicited a larger increase in renal sympathetic nerve activity compared to the response of each hormone alone. The high fat diet had no effect on the magnitude of the response by the end of the observation period when compared to rats fed a normal diet (Figure 3). Since the cellular transduction pathway mediating the renal sympathetic nerve responses to each hormone involves PI 3-Kinase, these studies suggest that a moderate high fat diet does not markedly influence the transduction pathway and the resultant efferent outflow. Thus, with respect to renal sympathetic nerve activity, central crosstalk between leptin and resistin occurred in rats fed a moderate high fat diet and the sensitivity of the interaction was not affected by the high fat diet.

### Crosstalk and Metabolic Regulation

There is a very limited number of studies that have investigated central crosstalk between leptin and resistin with respect to metabolic function. The potential for such an interaction is high. For example, and as indicated earlier in this review, leptin administered centrally elicits increases in sympathetic nerve activity to brown adipose tissue (Rahmouni et al., 2009). In contrast, central administration of resistin decreases sympathetic nerve activity to brown adipose tissue resulting in a reduced thermogenesis and reduced body core temperature (Kosari et al., 2011, 2013). Thus, when both hormones are elevated, the influence on sympathetic nerve activity to adipose tissue and thermogenesis would be expected to be the result of two opposing actions. This has not been investigated directly (Table 1). However, such an interaction is supported by the findings that show a decrease in the number of activated neurons in the raphe pallidus, a medullary brain nucleus that has a key role in regulating sympathetic nerve activity to brown adipose tissue (Morrison et al., 1999). Additionally, resistin can induce resistance to leptin’s thermogenic effects (Asterholm et al., 2014), which further suggests that central crosstalk between those two hormones has functional consequences on energy expenditure.

**TABLE 1:** Graphical representation of the effects of leptin, resistin and the combination of both administered centrally highlighting the crosstalk/interactions observed when leptin and resistin are present together.

Variable	Leptin	Resistin	Leptin + Resistin
**Metabolic**			
BAT SNA	↑	↓	?
Food Intake	↓↓	↓ (acute)/−(Chronic)	-
Insulin Secretion	↓↓	↑	↓
**Cardiovascular**			
Renal SNA	↑	↑	↑↑

*BAT, brown adipose tissue; SNA, sympathetic nerve activity. Arrows indicate direction and relative magnitude of change.*

Not only is there crosstalk on energy expenditure, leptin and resistin may interact centrally to affect dietary intake and thus body mass. Leptin is a potent anorectic whilst resistin may also reduce food intake acutely. Chronically, however, the decrease in food intake is not sustained. Furthermore, the chronic administration of leptin together with resistin intracerebroventricularly resulted in attenuation of the effects of chronic central administration of leptin on food consumption (Park et al., 2008) (Table 1). This crosstalk may be mediated by opposing actions on the phosphorylation of STAT3 in the hypothalamus which is increased by leptin but decreased by resistin (Park et al., 2008) as well as changes in phosphorylation of AMPK and of Akt which are increased by leptin but unaffected by resistin alone (Park et al., 2008).

Glucose homeostasis can also be affected by chronically administered leptin and resistin acting within the brain. Leptin can increase peripheral insulin sensitivity but reduces glucose-stimulated insulin secretion by acting within the hypothalamus. Resistin, also acting in the hypothalamus increases insulin sensitivity (but to a much lesser extent than leptin) but reduces glucose-stimulated insulin secretion, in contrast to leptin. Thus, here too, is the potential for central crosstalk (Table 1). Indeed, when leptin and resistin combined were infused intracerebroventricularly, insulin secretion was less than that observed with leptin alone suggesting resistin was able to inhibit leptin’s actions (Park et al., 2008). When glucose uptake was measured, there was no difference between the effects of leptin and resistin either alone or in combination (Park et al., 2008). This suggests that the crosstalk of centrally acting leptin and resistin on glucose homeostasis was primarily due to actions on insulin secretion (Table 1).

### Functional Implications of Crosstalk

According to the World Health Organisation, there are approximately 1.9 billion adults over 18 years of age, 38 million children under 5 years of age and over 340 million children between 5-18 years of age who are overweight or obese (WHO, 2020). Of great concern are the findings that of the overweight/obese children under 5 years of age, about half are found in Asia and one-quarter are in Africa. The epidemic of overweight/obesity shows no sign of abatement with the worldwide prevalence of obesity tripling since 1975 (WHO, 2020).

**FIGURE 4:**
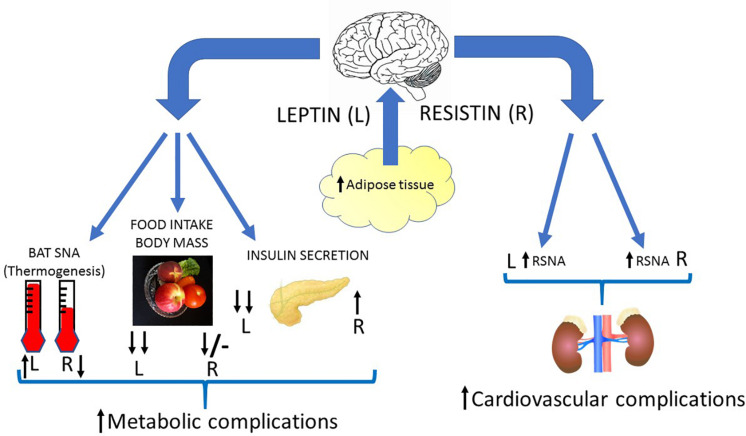
Diagrammatic depiction of the potential centrally mediated effects of increased leptin and resistin synthesis due to increased adiposity. Leptin and insulin can act in the brain to influence metabolic and cardiovascular outputs. Leptin elevates sympathetic nerve activity to the kidneys (RSNA), vasculature and to brown adipose tissue (BAT SNA). It also dramatically reduces food intake, body mass and decreases insulin secretion from the pancreas through actions in the brain. Resistin can act in the brain to increase sympathetic nerve activity to the kidneys and vasculature, but it decreases sympathetic nerve activity to brown adipose tissue. Resistin by acting in the brain increases insulin secretion and reduces food intake acutely (though less than leptin) and has little effect chronically. Thus, crosstalk within the brain between these two hormones could result in less thermogenesis (energy expenditure), a reduced anorexigenic effect, less insulin secretion but enhanced sympathetic nerve activity to the kidneys (and vasculature?) compared to the actions of leptin alone. This would result in increased metabolic and cardiovascular complications in conditions in which there is increased leptin and resistin. Black arrows signify direction and relative magnitude of change.

A characteristic of obesity, in both humans and in animal models, is an increase in sympathetic nerve activity to the kidneys (Grassi et al., 1998; Esler et al., 2006). This efferent output affects salt and water balance and plays an important role in hypertension associated with obesity (Kassab et al., 1995; Prior et al., 2014; Simonds et al., 2014). The causes of the elevated sympathetic nerve activity observed in obesity are unknown but leptin contributes (Lim et al., 2013). This could be important with respect to central crosstalk of leptin and resistin and the responses influencing cardiovascular regulation. As discussed in this review, the central crosstalk between leptin and resistin has been studied following acute administration of the two hormones into the cerebral ventricles and the findings show there is a marked enhancement in the sympatho-excitatory efferent output to the kidneys (Figure 4). Thus, it would seem likely that increased resistin levels could enhance the contribution of leptin on renal sympathetic nerve activity and result in worse cardiovascular complications.

The recent hypothesis that hyperleptinemia is the cause of leptin resistance in metabolic function (see below) has raised the possibility that partially reducing leptin levels increases sensitivity to leptin and this would influence the cardiovascular effects of leptin in high fat fed conditions. This has not been investigated to date, but if it were found to occur, it would suggest that the “selective leptin resistance” related to cardiovascular regulation in high fat fed conditions discussed earlier in this review, may need review and that partial reduction of leptin levels in the high fat fed conditions should see even greater cardiovascular responses to leptin. This is an intriguing suggestion and the implications for crosstalk with resistin may be even further cardiovascular complications. Clearly, this is an area for further investigation.

Although there are limited studies on the central crosstalk of leptin and resistin on metabolic function, the evidence indicates that the presence of resistin reduces the actions of leptin on food intake and it has opposing actions on insulin secretion and thermogenesis. In regard to food intake and insulin secretion, the crosstalk has been found in preclinical *in vivo* studies following chronic administration of leptin and resistin.

In recent work, evidence is suggesting that partially reducing the high leptin levels (20–80%) in rodents fed a high fat diet is sufficient to attenuate the leptin resistance and improve leptin sensitivity such that weight loss and insulin sensitivity and glucose homeostasis are improved from the hyperleptinemic condition (Zhao et al., 2019, 2020). It would be of great interest to determine the metabolic effects of central crosstalk between leptin and resistin in conditions in which leptin sensitivity are manipulated.

The opposing actions on thermogenesis, mediated by opposing changes in sympathetic nerve activity to brown adipose tissue have been observed following acute administration of leptin or resistin alone (Figure 4). If a similar effect occurred following chronic administration of the two hormones it would strongly suggest an important antagonistic interaction on energy expenditure. Such an interaction could be very important since there has been a dramatic increase in recent years in the potential of brown adipose tissue as a therapeutic target (Trayhurn, 2018). Clearly, increased sympathetic nerve activity to brown adipose tissue and increased thermogenesis resulting in increased energy expenditure would be beneficial in conditions of overweight/obesity. Thus, the ability of leptin to increase sympathetic nerve activity to brown adipose tissue would be a useful action. In the presence of hyperresistinemia the crosstalk between resistin and leptin would reduce leptin’s actions on sympathetic nerve activity to brown adipose tissue and would be detrimental in those conditions. It is noted that the role of leptin in thermogenesis and energy expenditure resulting in weight loss has been questioned recently though leptin’s ability to increase sympathetic nerve activity to brown adipose tissue is accepted (Fischer et al., 2020).

## Conclusion

The adipokines, resistin and leptin are produced in fat tissue and each can act in the brain to affect metabolic functions, like food intake, insulin sensitivity and release and energy homeostasis. They also influence cardiovascular regulation. Leptin and resistin alone increase sympathetic nerve activity to the vasculature and kidneys and their actions are enhanced, as shown by the larger excitatory response observed in renal sympathetic nerve activity, when both are present compared to their individual responses. These effects are mediated by actions within the hypothalamus indicating crosstalk, probably involving similar intracellular transductions pathways.

By contrast, metabolic actions of leptin and resistin alone can oppose each other. The central actions of leptin include decreases in food intake, decreases in insulin release and elevated thermogenesis mediated via increased sympathetic nerve activity to brown adipose tissue. The central actions of resistin elicits decreased food intake but increased insulin secretion and reduced thermogenesis. When resistin was combined with leptin, the magnitude of the decrease in food intake induced by leptin was reduced and the decrease in insulin secretion induced by leptin was reduced, suggesting the centrally mediated crosstalk between these two hormones may be detrimental to the metabolic regulation in conditions of hyperleptinemia and hyperresistinemia. The potential for crosstalk on the responses of leptin and resistin on sympathetic nerve activity innervating brown adipose tissue has not been investigated directly. However, given the clear opposing actions on this sympathetic outflow following acute administration of each hormone, we suspect that crosstalk between leptin and resistin on thermogenesis would also negatively impact energy homeostasis.

The evidence, therefore, suggests that in conditions of hyperleptinemia and hyperresistinemia, such as overweight, obesity or metabolic syndrome, the potential for central crosstalk between leptin and resistin is high and the result could contribute to worsened metabolic function and cardiovascular dysfunction (Figure 4). We suspect that this crosstalk is not restricted to these two adipokines alone but may be more widespread and is an area that needs further investigation.

## Author Contributions

EB wrote the review and designed the figures.

## Conflict of Interest

The authors declare that the research was conducted in the absence of any commercial or financial relationships that could be construed as a potential conflict of interest.
